# Usefulness of combining serum uric acid and high-sensitivity C-reactive protein for risk stratification of patients with metabolic syndrome in community-dwelling women

**DOI:** 10.1007/s12020-013-9912-3

**Published:** 2013-03-09

**Authors:** Ryuichi Kawamoto, Yasuharu Tabara, Katsuhiko Kohara, Tetsuro Miki, Tomo Kusunoki, Shuzo Takayama, Masanori Abe, Tateaki Katoh, Nobuyuki Ohtsuka

**Affiliations:** 1 Department of Community Medicine, Ehime University Graduate School of Medicine, Ehime, 791-0295 Japan; 2 Department of Geriatric Medicine, Ehime University Graduate School of Medicine, Ehime, 791-0295 Japan; 3 Department of Internal Medicine, Seiyo Municipal Nomura Hospital, 9-53 Nomura, Nomura-cho, Seiyo-city, Ehime 797-1212 Japan

**Keywords:** Uric acid, C-reactive protein, Metabolic syndrome, Insulin resistance, Women

## Abstract

Metabolic syndrome (MetS) is associated with an increased risk of major cardiovascular events. In women, increased uric acid (UA) levels are associated with MetS and its components. High-sensitivity C-reactive protein (hsCRP) levels are also associated with MetS, and hsCRP levels could be modulated by UA. We investigated whether combining UA and hsCRP levels are independently associated with MetS and insulin resistance in Japanese community-dwelling women. From a single community, we recruited 1,097 women (63 ± 12 years) during their annual health examination, and examined the cross-sectional relationship between UA, hsCRP, and MetS and insulin resistance, which was evaluated by homeostasis of minimal assessment of insulin resistance. Of these subjects, 218 women (19.9 %) had MetS. Multiple linear regression analysis was performed to evaluate the contribution of each confounding factor for MetS and insulin resistance, both UA and hsCRP as well as age and alcohol consumption, were independently and significantly associated with MetS and insulin resistance. The adjusted-odds ratios (95 % confidence interval) for MetS across tertiles of UA and hsCRP were 1.00, 1.45 (0.95–2.22), and 2.61 (1.74–3.93), and 1.00, 1.80 (1.18–2.74), and 3.23 (2.15–4.85), respectively. In addition, the combination increased UA, and hsCRP was also a significant and independent determinant for MetS and insulin resistance. In direction associations, we also observed a synergistic effect between these two molecules (*F* = 2.76, *P* = 0.027). These results suggested that combined assessment of UA and hsCRP levels provides incremental information for risk stratification of patients with MetS, independent of other confounding factors in community-dwelling women.

## Introduction

Metabolic syndrome (MetS), a clustering of cardiovascular risk factors such as visceral obesity, insulin resistance, hypertension, glucose intolerance, hypertriglyceridemia, and low high-density lipoprotein cholesterol (HDL-C) levels, is a major worldwide public health problem. In Japan, it is 13.3 % in men and 11.5 % in women [1]. MetS increases the risk of diabetes, atherosclerotic disease [2], and cardiovascular disease (CVD) [3]. With the continuous increase in obesity prevalence in Japan, MetS may become even more common.

Recently, results from a meta-analysis of individual participant data from 54 long-term prospective studies demonstrated that high-sensitivity C-reactive protein (hsCRP) is also an inflammatory marker and independent predictor of CVD such as coronary heart disease, ischemic stroke, and vascular and non-vascular mortality [4]. Several studies have demonstrated that many people with MetS have an elevated hsCRP concentration [5], which may predict their risk for future adverse events [6].

Increased serum uric acid (UA) in humans is also associated with systemic inflammation [7], endothelial dysfunction [8], hypertension [9], CVD, and CVD mortality [10]. Despite a strong association between serum UA level and various CVD in humans, UA is not considered as having a pathogenetic role in these conditions, and instead, is considered as biologically inert or possibly anti-inflammatory because it could function as an antioxidant [11]. However, Koga et al. [12] reported that UA showed a significant correlation with hsCRP, suggesting a possible effect between these two key markers. Furthermore, UA is more strongly associated with MetS in women than in men [13]. However, there are few reports on the relationship between combining UA and CRP, and MetS in Japanese women.

The aim of this study was to determine whether combining UA and hsCRP levels are independently associated with MetS and insulin resistance by examining cross-sectional data from Japanese community-dwelling women.

## Methods

### Subjects

Participants were recruited at the time of their annual health examination in a rural town with a total population of 11,136 (as of April 2002) and located in Ehime prefecture, Japan, in 2002. Among the 4,738 female aged 19–90 years in this population, a random sample of 1,713 (36.2 %) subjects was recruited. For all these individuals, overnight fasting plasma sample were available for measuring hsCRP. The final study sample compromised 1,097 residents. This study was approved by the ethics committee of Ehime University School of Medicine, and written informed consent was obtained from each subjects.

### Evaluation of risk factors

Information on demographic characteristics and risk factors was collected using the clinical files. Body mass index was calculated by dividing weight (in kg) by the square of the height (in m). We measured blood pressure with an appropriate-sized cuff on the right upper arm of the subjects in the sedentary position using an automatic oscillometric blood pressure recorder (BP-103i; Colin, Aichi, Japan) while they were seated after having rested for at least 5 min. Other characteristics such as smoking, alcohol habit, and medication, were investigated by individual interviews that were conducted using a structured questionnaire. Smoking status was classified into non-current smoker and current smokers. The daily alcohol consumption was measured using the Japanese liquor unit in which a unit corresponds to 22.9 g of ethanol, and the participants were classified into never-drinkers, occasional drinkers (<1 U/day), and light–heavy drinkers (≥1 U/day). Total cholesterol (T-C), triglycerides (TG), HDL-C, fasting plasma glucose (FPG), and UA were measured by the laboratory of health examination center during an overnight fast of more than 11 h. The plasma sample were immediately frozen and stored at −80 °C until measurements were taken by the laboratory of our department. The plasma hsCRP concentration was measured using a Behring BN II nephelometer (Dade Behring Inc., Marburg, Germany) and the inter- and intra-assay coefficient variations were 3.2 and 6.7 %, respectively. Low-density lipoprotein cholesterol (LDL-C) level was calculated by the Friedewald formula [14]. Individuals with TG levels ≥400 mg/dL were excluded. Homeostasis of model assessment of insulin resistance (HOMA-IR) was calculated from FPG and IRI levels using the following formula: [FPG (mg/dL) × IRI (mU/mL)]/405 [15]. Insulin resistance was defined as a HOMA-IR ≥2.6 [16].

### Metabolic syndrome

We applied condition-specific cutoff points for MetS based on the modified criteria of the National Cholesterol Education Program’s Adult Treatment Panel (NCEP-ATP) II report [17]. MetS was defined as subjects with at least three or more of the following five conditions: (1) obesity: BMI ≥25.0 kg/m^2^ according to the guidelines of the Japanese Society for the Study of Obesity (waist circumference was not available in this study) [18]; (2) raised BP with a systolic blood pressure (SBP) ≥130 mmHg and/or diastolic blood pressure (DBP) ≥85 mmHg, and/or current treatment for hypertension; (3) Hypertriglyceridemia with a TG level ≥150 mg/dL; (4) low HDL cholesterolemia with a HDL-C <50 mg/dL in women and/or current treatment for dyslipidemia; and (5) impaired fasting glucose with a FPG level ≥100 mg/dL and/or current treatment for diabetes mellitus.

### Statistical analysis

**Fig. 1:**
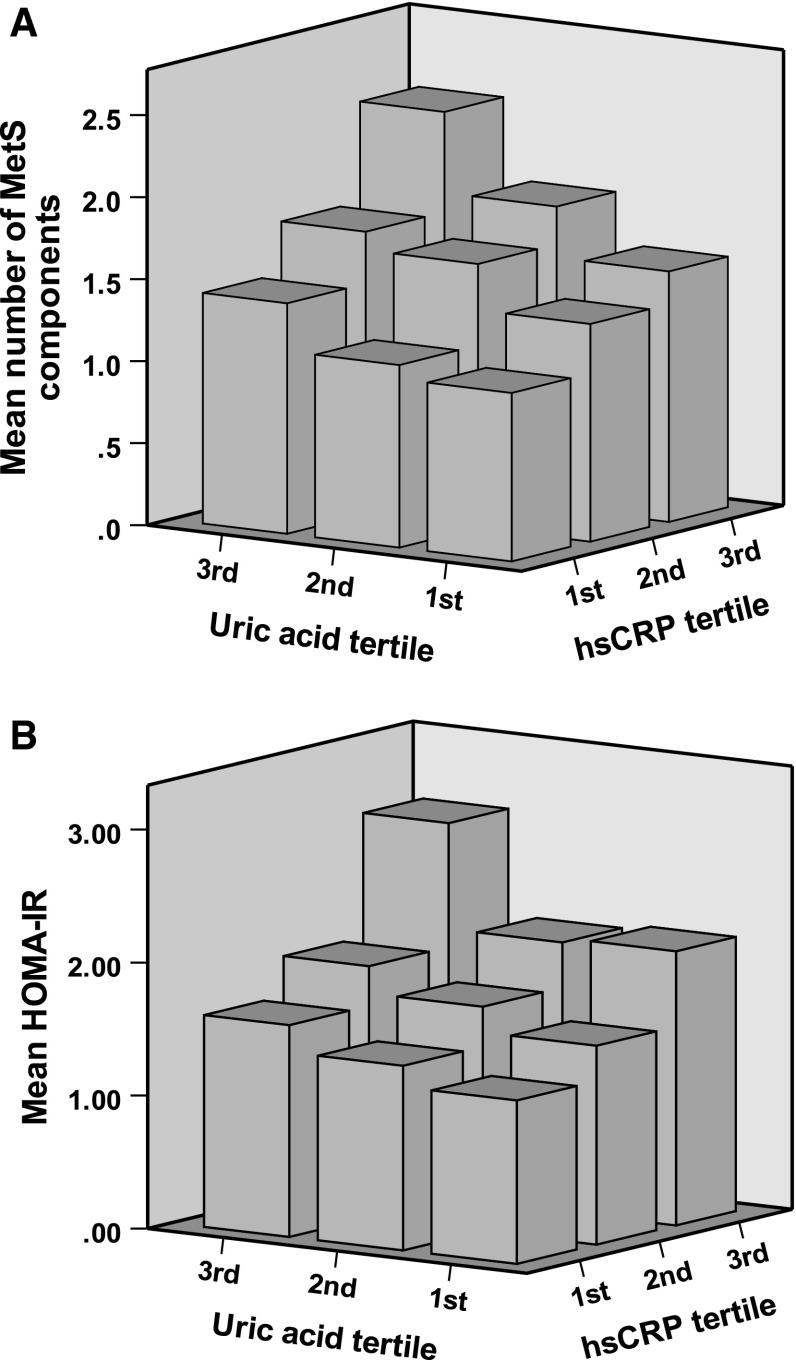
Combining effect of UA and hsCRP. **a** Mean accumulating number of metabolic syndrome (MetS) components: obesity, raised blood pressure, hypertriglyceridemia, low HDL cholesterolemia, and impaired fasting glucose. **b** HOMA-IR. Study subjects were divided into three groups (tertiles) according to the UA and hsCRP levels

Data are presented as the mean ± standard deviation (SD) unless otherwise specified, and in the cases of parameters with non-normal distributions (TG, FPG, HOMA-IR, and hsCRP), the data are shown as median (interquartile range) values. In all analyses, parameters with non-normal distributions were used after log-transformation. Statistical analysis was performed using PASW Statistics 17.0 (Statistical Package for Social Science Japan, Inc., Tokyo, Japan). Correlations between UA and hsCRP were determined using Pearson’s correlation. Multiple linear regression analysis was used to evaluate the contribution of confounding factors (e.g., age, alcohol consumption, current smoking status, UA, and hsCRP) for number of MetS components and HOMA-IR. Subjects were divided into three groups based on tertiles of UA and hsCRP, and differences among the groups categorized by each tertile of UA and hsCRP were analyzed by ANOVA for continuous variables or the *χ* ^2^ test for categorical variables. Multiple logistic regression analyses were used to evaluate the contribution of confounding factors for MetS and insulin resistance (HOMA-IR ≥ 2.6), and further used to also evaluate the contribution of each tertile of UA and hsCRP for MetS, each component of MetS, and insulin resistance. The combining effect of UA and hsCRP was evaluated using multiple logistic regression analyses adjusted for the following parameters: age, current smoking status, alcohol consumption, UA, and hsCRP. The synergistic effect of UA and hsCRP was evaluated using a general linear model (Fig. 1). A *P* value <0.05 was considered significant.

## Results

### Characteristics of subjects

The characteristics of the subjects categorized according to tertiles of UA and hsCRP are illustrated in Table 1. The study included 1,097 women, aged 63 ± 12 (range 21–88) years. Age, BMI, SBP, DBP, the presence of antihypertensive medication, TG, LDL-C, FPG, and HOMA-IR were significantly higher in relation to the higher tertiles of UA and hsCRP, but HDL-C were significantly lower in the higher tertiles. Alcohol consumption increased significantly in correlation with an increase in tertile of UA, and history of CVD increased with tertile of hsCRP. There was no inter-group difference in current smoking status, and the presence of antilipidemic and antidiabetic medication.

**Table 1:** Characteristics of subjects categorized according to tertiles of uric acid and hsCRP

Characteristics	Tertile of uric acid				Tertile of hsCRP			
	1st *N* = 366	2nd *N* = 379	3rd *N* = 352	*P* value	1st *N* = 414	2nd *N* = 353	3rd *N* = 330	*P* value
	<4.0	4.0–4.8	>4.8 mg/dL		<0.032	0.032–0.071	>0.071 mg/dL	
Age (years)	62 ± 12	62 ± 12	65 ± 11	0.003	60 ± 13	64 ± 11	65 ± 10	<0.001
Body mass index (kg/m^2^)	22.3 ± 3.0	23.4 ± 3.1	24.6 ± 3.6	<0.001	21.9 ± 2.8	23.6 ± 3.0	25.0 ± 3.6	<0.001
Current smoking status (%)^a^	0.8	1.6	2.6	0.186	1.7	1.4	1.8	0.914
Alcohol consumption (%)^b^	69.1/28.7/2.2	62.8/31.4/5.8	61.9/29.8/8.2	0.006	60.1/32.6/7.2	66.3/28.9/4.8	68.5/27.9/3.6	0.074
History of CVD (%)	5.5	6.6	8.5	0.261	4.8	5.9	10.3	0.010
Systolic blood pressure (mmHg)	135 ± 23	138 ± 23	143 ± 23	<0.001	135 ± 23	140 ± 23	142 ± 23	<0.001
Diastolic blood pressure (mmHg)	78 ± 12	80 ± 11	82 ± 12	<0.001	79 ± 12	81 ± 12	81 ± 11	<0.001
Antihypertensive medication (%)	14.8	26.6	35.8	<0.001	17.9	27.8	33.0	<0.001
Triglycerides (mg/dL)	80 (61–107)	89 (67–119)	105 (76–144)	<0.001	83 (60–111)	91 (70–125)	100 (74–144)	<0.001
HDL cholesterol (mg/dL)	67 ± 15	65 ± 16	63 ± 16	0.005	68 ± 16	64 ± 15	61 ± 15	<0.001
LDL cholesterol (mg/dL)	121 ± 29	126 ± 30	131 ± 30	<0.001	118 ± 28	130 ± 29	132 ± 30	<0.001
Antilipidemic medication (%)	5.5	6.9	7.7	0.486	4.8	6.8	8.8	0.098
Fasting plasma glucose (mg/dL)	91 (86–98)	92 (87–99)	95 (89–102)	0.003	90 (85–97)	92 (87–100)	95 (89–104)	<0.001
HOMA-IR	1.22 (0.76–1.72)	1.35 (0.94–1.95)	1.71 (1.17–2.61)	<0.001	1.14 (0.77–1.63)	1.44 (0.99–2.09)	1.70 (1.14–2.82)	<0.001
Antidiabetic medication (%)	3.6	2.4	3.7	0.533	1.7	3.4	4.8	0.050
Uric acid (mg/dL)	3.4 ± 0.5	4.4 ± 0.2	5.7 ± 0.7	<0.001	4.1 ± 0.9	4.5 ± 1.0	4.8 ± 1.1	<0.001
hsCRP (mg/dL)	0.031 (0.016–0.060)	0.042 (0.022–0.077)	0.064 (0.032–0.124)	<0.001	0.018 (0.012–0.024)	0.047 (0.038–0.059)	0.135 (0.094–0.294)	<0.001

Data presented are mean ± standard deviation. Data for triglycerides, fasting plasma glucose, HOMA-IR, and hsCRP were skewed and are presented as median (interquartile range) values, and were log-transformed for analysis

*hsCRP* high-sensitivity C-reactive protein, *CVD* cardiovascular disease, *HDL* high-density lipoprotein, *LDL* low-density lipoprotein, *HOMA-IR* homeostasis of model assessment of insulin resistance

* *P* value from ANOVA for continuous variables or from *χ* ^2^ test for categorical variables

^a^Current smoking status was classified into non-current smoker and current smokers

^b^Alcohol consumption was measured using a Japanese liquor unit where 1 U corresponds to 22.9 g of ethanol [never-drinkers, occasional drinkers (<1 U/day), and light-heavy drinkers (≥1 U/day)]

### Association between various characteristics, and MetS and insulin resistance

UA levels significantly increased with hsCRP (*r* = 0.285, *P* < 0.001; data not shown). As shown in Table 2, multiple linear regression analysis was used to evaluate the contribution of each confounding factor for MetS and insulin resistance. Both UA and hsCRP as well as age and alcohol consumption were independently significantly associated with number of MetS, and both UA and hsCRP was associated with insulin resistance. In addition, after adjustment for history of CVD and medication (i.e., antihypertensive, antilipidemic, and antidiabetic medication), these results were similar.

**Table 2:** Multiple linear regression analysis for number of MetS components and HOMA-IR

Characteristics	Number of MetS components		HOMA-IR	
	*β* (*P* value)	*β* (*P* value)	*β* (*P* value)	*β* (*P* value)
Age (years)	0.240 (<0.001)	0.230 (<0.001)	0.019 (0.541)	−0.031 (0.333)
Current smoking status, *N* (%)	−0.011 (0.701)	−0.012 (0.676)	−0.006 (0.846)	−0.004 (0.887)
Alcohol consumption, *N* (%)	−0.068 (0.020)	−0.066 (0.025)	−0.044 (0.148)	−0.032 (0.293)
History of CVD	–	0.060 (0.032)	–	0.000 (0.991)
Medication	–	–	–	0.170 (<0.001)
Uric acid (mg/dL)	0.189 (<0.001)	0.188 (<0.001)	0.190 (<0.001)	0.161 (<0.001)
hsCRP (mg/dL)	0.180 (<0.001)	0.178 (<0.001)	0.229 (<0.001)	0.220 (<0.001)
*R* ^2^	0.187 (<0.001)	0.191 (<0.001)	0.119 (<0.001)	0.143 (<0.001)

Metabolic syndrome (MetS) components were defined as the following conditions: obesity, raised blood pressure, hypertriglyceridemia, low HDL cholesterolemia, and impaired fasting plasma glucose. Medications include antihypertensive, antilipidemic, and antidiabetic medication

### Prevalence and adjusted-odds ratio for MetS, its components, and insulin resistance in relation to tertiles of UA and hsCRP

Table 3 shows prevalence and the risk for MetS and abnormalities of its components in relation to tertiles of UA and hsCRP among 1,097 women. Of these, 218 women (19.9 %) had MetS. As shown in Table 3, after adjustments for age, current smoking status, and alcohol consumption, the prevalence rate of MetS, its components, and insulin resistance increased significantly in relation to the higher tertiles of UA and hsCRP. The ORs (95 % CI) for MetS across the tertiles of UA and hsCRP were 1.00, 1.45 (0.95–2.22), and 2.61 (1.74–3.93), and 1.00, 1.80 (1.18–2.74), and 3.23 (2.15–4.85), respectively. The ORs of UA were significantly high for the MetS components of obesity, raised blood pressure, hypertriglyceridemia, and ORs of hsCRP were significantly high for obesity, hypertriglyceridemia, low HDL cholesterolemia, and impaired fasting glucose. The ORs for insulin resistance also increased significantly in relation to both UA and hsCRP.

**Table 3:** The prevalence and adjusted ORs for MetS, its components, and insulin resistance in relation to the tertiles of uric acid and hsCRP

Characteristics	Tertile of uric acid				Tertile of hsCRP			
	1st *N* = 366	2nd *N* = 379	3rd *N* = 352	*P* value	1st *N* = 414	2nd *N* = 353	3rd *N* = 330	*P* value
	<4.0	4.0–4.8	>4.8 mg/dL		<0.032	0.032–0.071	>0.071 mg/dL	
MetS	43 (11.7 %)	66 (17.4 %)	109 (31.0 %)	<0.001	42 (10.1 %)	68 (19.3 %)	108 (32.7 %)	<0.001
Adjusted OR (95 % CI)	Reference	1.45 (0.95–2.22)	2.61 (1.74–3.93)	<0.001	Reference	1.80 (1.18–2.74)	3.23 (2.15–4.85)	<0.001
Components of MetS								
Obesity	63 (17.2 %)	105 (27.7 %)	157 (44.6 %)	<0.001	62 (15.0 %)	103 (29.2 %)	160 (48.5 %)	<0.001
Adjusted OR (95 % CI)	Reference	1.63 (1.13–2.35)	2.85 (1.98–4.10)	<0.001	Reference	2.15 (1.49–3.09)	4.55 (3.17–6.54)	<0.001
Raised blood pressure	219 (59.8 %)	253 (66.8 %)	261 (74.1 %)	<0.001	249 (60.1 %)	244 (69.1 %)	240 (72.7 %)	0.001
Adjusted OR (95 % CI)	Reference	1.41 (0.99–1.98)	1.75 (1.20–2.55)	0.012	Reference	1.06 (0.75–1.50)	1.05 (0.72–1.51)	0.945
Hypertriglyceridemia	32 (8.7 %)	47 (12.4 %)	81 (23.0 %)	<0.001	37 (8.9 %)	49 (13.9 %)	74 (22.4 %)	<0.001
Adjusted OR (95 % CI)	Reference	1.41 (0.87–2.28)	2.61 (1.65–4.12)	<0.001	Reference	1.36 (0.86–2.17)	2.12 (1.36–3.31)	0.003
Low HDL cholesterolemia	64 (17.5)	91 (24.0)	86 (24.4)	0.040	66 (15.9 %)	78 (22.1 %)	97 (29.4 %)	<0.001
Adjusted OR (95 % CI)	Reference	1.44 (1.00–2.08)	1.31 (0.89–1.94)	0.132	Reference	1.35 (0.93–1.95)	1.93 (1.33–2.79)	0.002
Impaired fasting glucose	82 (22.4 %)	91 (24.0 %)	113 (32.1 %)	0.007	75 (18.1 %)	97 (27.5 %)	114 (34.5 %)	<0.001
Adjusted OR (95 % CI)	Reference	1.01 (0.71–1.44)	1.30 (0.91–1.85)	0.253	Reference	1.51 (1.06–2.15)	1.93 (1.35–2.75)	0.001
Insulin resistance^a^	36 (9.8 %)	47 (12.4 %)	90 (25.6 %)	<0.001	31 (7.5 %)	49 (13.9 %)	93 (28.2 %)	<0.001
Adjusted OR (95 % CI)	Reference	1.14 (0.71–1.83)	2.28 (1.47–3.55)	<0.001	Reference	1.14 (0.71–1.83)	2.28 (1.47–3.55)	<0.001

Study subjects were divided into three groups (tertiles) according to uric acid and hsCRP levels. Adjusted for age, current smoking status, and alcohol consumption

*OR* odds ratio, *CI* confidence interval

^a^Insulin resistance was defined as HOMA-IR ≥2.6

### Combining effects of UA and hsCRP on mean accumulating number of MetS components and insulin resistance

We assessed the statistical significance of the combining relationship using multiple logistic regression analyses adjusted for the confounding factors (Table 4). The odds ratio of an increased tertile of UA and hsCRP was a significant and independent determinant for MetS and insulin resistance.

**Table 4:** Multiple logistic regression analysis for MetS and insulin resistance

Characteristics	MetS adjusted OR (95 % CI)			Insulin resistance^a^ adjusted OR (95 % CI)		
	hsCRP-1st	hsCRP-2nd	hsCRP-3rd	hsCRP-1st	hsCRP-2nd	hsCRP-3rd
Uric acid-1st	Reference	1.26 (0.57–2.87)	3.31 (1.51–7.24)	Reference	1.22 (0.52–2.87)	1.48 (0.58–3.80)
Uric acid-2nd	1.93 (0.87–4.30)	3.31 (1.64–6.68)	3.89 (1.89–8.01)	1.70 (0.72–4.01)	1.91 (0.87–4.19)	3.56 (1.68–7.58)
Uric acid-3rd	3.36 (1.52–7.43)	4.35 (2.11–8.99)	9.25 (4.81–17.8)	3.11 (1.34–7.21)	3.60 (1.66–7.77)	8.92 (4.54–17.6)

Metabolic syndrome (MetS) was defined as having three or more of the following conditions: obesity, raised blood pressure, hypertriglyceridemia, low HDL cholesterolemia, and impaired fasting plasma glucose. Adjusted for the following parameters: age, current smoking status, and alcohol consumption

^a^Insulin resistance was defined as HOMA-IR ≥2.6

### Synergistic effects of UA and hsCRP

In addition to their direct associations, we observed a synergistic effect between UA and hsCRP. In Fig. 1, subjects were divided into three groups according to the tertiles of UA and hsCRP levels. We assessed the statistical significance of the synergistic relationship using a general linear model with the following confounding factors: age, current smoking, and alcohol consumption. The interaction between increased UA and hsCRP was a significant and independent determinant for the accumulation of MetS components (*F* = 2.76, *P* = 0.027), in addition to their direct associations (UA tertile, *F* = 18.6, *P* < 0.001; hsCRP tertile, *F* = 26.1, *P* < 0.001).

## Discussion

In 1,097 community-dwelling women, we determined the prevalence rate of MetS, as defined by the modified NCEP-ATP III criteria [17], and examined the association between UA and hsCRP, and MetS and its components. MetS was common, occurring in 19.9 % of women. In women, the prevalence rate of MetS increased significantly in relation to UA and hsCRP, even after adjusting for age, smoking status, and alcohol consumption, and the ORs of MetS increased dose dependently with increasing tertiles of UA and hsCRP. In addition, we demonstrated that the ORs of MetS and insulin resistance were significantly increased in relation to combining UA and hsCRP. To our knowledge, this is the first study to indicate these associations of UA and hsCRP with MetS and insulin resistance in about 1,000 community-dwelling women.

Elevated CRP was associated with increased odds of MetS after adjusting for potential confounding factors [19]. In a rural Chinese population, the prevalence of MetS increased progressively with elevated CRP levels (*P* < 0.001 for trend). In the highest quartile of CRP levels (>1.50 mg/L), the risk for MetS was substantially higher (0R 5.97; 95 % CI 4.75–7.51) compared with that in the lowest quartile of CRP levels (≤0.33 mg/L) after adjustment for age, sex, geographic location, lifestyle factors, level of education attained, and family history of chronic diseases [19]. We have also reported that after adjusting for age, smoking status, LDL-C, creatinine and history of diabetes mellitus, the ORs (95 % CI) of the sex-specific quartiles of UA for MetS were 1.0, 1.04 (0.56–1.94), 2.35 (1.30–4.22), and 2.20 (1.16–4.20) in women [13]. In this study, increased UA and hsCRP were independently and significantly associated with the prevalence of MetS and insulin resistance.

The mechanisms by which UA reflect the risk for MetS are not completely understood. In the past, most authorities have not considered UA as having a pathogenetic role of CVD and have viewed UA as biologically inert or possibly anti-inflammatory because it could function as an antioxidant [11]. However, recent studies have challenged this viewpoint and it has been reported that both elevated UA may also reflect systemic inflammation [20] and is closely associated with the pathogenesis of MetS and type 2 diabetes [21] which impairs insulin signaling in the liver, muscle, and adipose tissues [22], and carotid intima-media thickness which is a surrogate maker [23]. Kang et al. [24] demonstrated that UA-induced changes in vascular proliferation and function may be mediated by de novo production of CRP in human vascular cells. Nakagawa et al. [25] recently reported that UA reduces levels of endothelial nitric oxide (NO) which is a key mediator of insulin action and increases blood flow to skeletal muscle and enhances glucose uptake. In addition, UA involves activation of the renin angiotensin system [26] and direct actions on endothelial and vascular smooth muscle cells [24]. Moreover, UA was also found to have a significant causal role in MetS that was induced experimentally by fructose [27]. Excessive fructose intake induces hyperuricemia and features of MetS, such as an increase in ambulatory BP and TG, but a decrease in HDL-C in healthy men [27, 28]. Perez-Pozo et al. [28] showed that lowering UA by allopurinol treatment prevented the increase in ambulatory BP.

There are some limitations to this study. First, our cross-sectional study design does not eliminate potential causal relationships between UA, hsCRP, and MetS. Second, the prevalence rate of MetS, UA, and hsCRP categories is based on a single assessment of blood, which may introduce a misclassification bias. Third, we used BMI ≥25 to classify individuals with visceral obesity because waist circumference measurements were not available, which might have caused an under or over estimation of the effect of visceral obesity on MetS [29]. In fact, the prevalence rate of MetS in women was higher than those generally reported for Japanese [30]. Fourth, we could not eliminate possible effects of the underlying diseases, and medication, especially diuretic use and antilipidemic drug use, on the results. These points need to be addressed again in a large population-based sample in a prospective manner.

In conclusion, the present study showed that combining UA and hsCRP levels are significantly associated with MetS, independent of other confounding factors in community-dwelling women. The underlying mechanism behind this relationship is unclear, but seems to be independent of traditional cardiovascular risk factors such as age, current smoking status, and alcohol consumption. For community-dwelling healthy women, prospective population-based studies are needed to investigate the mechanisms underlying this association to determine whether intervention, such as effective lifestyle modifications or medication (e.g., antihypertensive, antilipidemic, and diabetic medication) that decrease UA and hsCRP in adults [31], will decrease the risk of MetS.
